# Hem1 inborn errors of immunity: waving goodbye to coordinated immunity in mice and humans

**DOI:** 10.3389/fimmu.2024.1402139

**Published:** 2024-07-04

**Authors:** Alexandra Christodoulou, Julia Y. Tsai, Nutthakarn Suwankitwat, Andreas Anderson, Brian M. Iritani

**Affiliations:** ^1^ The Department of Comparative Medicine, University of Washington, Seattle, WA, United States; ^2^ Virology Laboratory, National Institute of Animal Health, Bangkok, Thailand

**Keywords:** Hem1, hematopoietic protein-1, wave, immunodeficiency disease, actin cytoskeleton, NCKAP1L, inborn errors of immunity, Hem-1

## Abstract

Inborn errors of immunity (IEI) are a group of diseases in humans that typically present as increased susceptibility to infections, autoimmunity, hyperinflammation, allergy, and in some cases malignancy. Among newly identified genes linked to IEIs include 3 independent reports of 9 individuals from 7 independent kindreds with severe primary immunodeficiency disease (PID) and autoimmunity due to loss-of-function mutations in the *NCKAP1L* gene encoding Hematopoietic protein 1 (HEM1). HEM1 is a hematopoietic cell specific component of the WASp family verprolin homologous (WAVE) regulatory complex (WRC), which acts downstream of multiple immune receptors to stimulate actin nucleation and polymerization of filamentous actin (F-actin). The polymerization and branching of F-actin is critical for creating force-generating cytoskeletal structures which drive most active cellular processes including migration, adhesion, immune synapse formation, and phagocytosis. Branched actin networks at the cell cortex have also been implicated in acting as a barrier to regulate inappropriate vesicle (e.g. cytokine) secretion and spontaneous antigen receptor crosslinking. Given the importance of the actin cytoskeleton in most or all hematopoietic cells, it is not surprising that HEM1 deficient children present with a complex clinical picture that involves overlapping features of immunodeficiency and autoimmunity. In this review, we will provide an overview of what is known about the molecular and cellular functions of HEM1 and the WRC in immune and other cells. We will describe the common clinicopathological features and immunophenotypes of HEM1 deficiency in humans and provide detailed comparative descriptions of what has been learned about Hem1 disruption using constitutive and immune cell-specific mouse knockout models. Finally, we discuss future perspectives and important areas for investigation regarding HEM1 and the WRC.

## Introduction

Effective immunity requires that multiple immune cells work synergistically to sense and engulf foreign invaders, process and present antigens to fellow immune cells which then clonally expand, adhere to the vascular endothelium, migrate to sites of infection, and release vesicles containing activating and inhibitory cytokines that coordinate recruitment, activation, and cessation of the immune response. Central to these dynamic processes is the coordinated and directional polymerization and depolymerization of filamentous actin (F-actin), which stimulates active branching networks of actin filaments that drive force generating structures. In immune cells, F-actin polymerization is initiated by receptor-ligand interactions which result in activation of two critical actin-regulatory complexes called WASp (Wiskott-Aldrich Syndrome protein) and the WAVE (WASP-family verprolin homologous protein) regulatory complex (WRC). Activation of the WASp and WRC culminate in activation of the actin related proteins 2/3 (ARP2/3) complex, which builds new F-actin filaments from existing ones. The importance of properly regulated polymerization and depolymerization of F-actin is underscored by the identification of approximately 24 inborn errors of immunity (IEI) in humans (“actinopathies”) to date caused by variants in genes encoding proteins involved in the regulation of the actin cytoskeleton in immune cells [reviewed in (1–4)]. The clinical and cellular consequences of actinopathies in humans tend to be unique due to the complexity and number of proteins involved in actin regulation, differences in tissue expression, distinctive biological functions, and surprisingly specific molecular and cellular roles of actin regulators.

Here, we will review a recently identified IEI caused by loss-of-function variants in the *NCKAP1L* gene encoding Hematopoietic Protein-1 (HEM1), a hematopoietic cell specific component of the WRC. We will provide an overview of what is known about the molecular and cellular regulation of the WRC and HEM1 in immune and other cells. We will describe what has been learned about the consequences of Hem1 deficiency in humans and murine models with a focus on the consequences of cell type specific Hem1 disruption.

## Activation of WASp and WAVE signaling complexes and actin polymerization by the Rho-family GTPases

**Figure 1:**
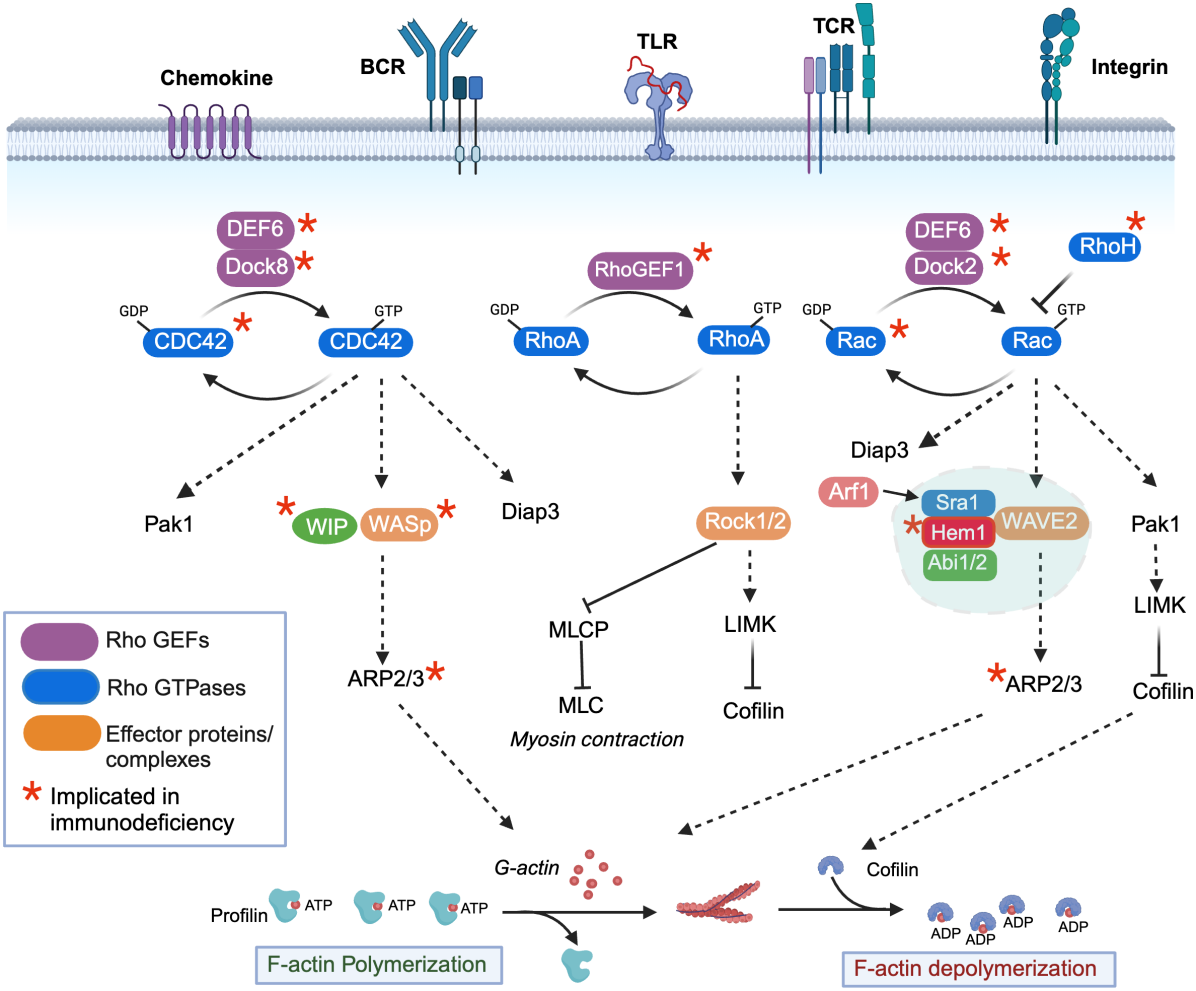
Regulation of filamentous actin polymerization by the rho-family GTPases, Hem1, and the WAVE regulatory complex. Activation of immune receptors upon ligand binding results in activation of Rho guanine nucleotide exchange factors (Rho-GEFs), which catalyze the release of GDP for GTP on Rho-GTPases (CDC42, RhoA, Rac1/2/3), resulting in GTPase activation. Conversely, Rho-GTPase activating proteins (Rho-GAPs) cause GTP to be hydrolyzed back to the inactive GDP-bound form. CDC42-GTP specifically binds and activates the WIP-WASp complex (*left*), which facilitates ARP2/3-mediated nucleation of ATP-bound G-actin, resulting in its release from Profilin and polymerization into filamentous actin (F-actin). GTP-bound Rac (*right*) predominantly interacts with and activates the WAVE regulatory complex (WRC), which also facilitates ARP2/3 nucleation of ATP-G-actin into F-actin. The Arf1 GTPase interaction with Sra1 enhances WRC activation. Both CDC42-GTP and Rac-GTP activate Pak-dependent Lim kinase (Pak1), which stabilizes F-actin by preventing Cofilin from severing ADP-bound actin and promoting actin depolymerization, and Diap3, which is required for nucleation of branched actin filaments. RhoA-GTP (*middle*) controls dynamic aspects of actin regulation by activating Rho-associated coiled-coil-containing kinase (ROCK), which inhibits myosin light chain phosphatase (MCLP), leading to increased phosphorylation of myosin light chains (MLCs), which bind actin and stimulate contraction at the “tail” during migration. RhoA-GTP also activates LIMK. An additional atypical RhoGTPase RhoH that is not regulated by GTP exchange is also present in hematopoietic cells and it believed to act in part by inhibiting the membrane association and activation of Rac-GTP. *Denotes genes where variants have been associated with Inborn Errors of Immunity (IEI).

Branched actin networks are the major force generating structures that drive most active processes in metazoan cells. Not surprisingly, highly conserved signaling molecules and pathways have evolved to regulate actin dynamics in response to activating stimuli. Although there are many proteins involved in tuning different aspects of actin dynamics including elongation, stability, turnover, capping, and severing [ reviewed in (5, 6)], we will focus primarily on regulation of WASp and the WRC, which drive the core activities required for building actin filaments. At the heart of WASp and WRC activation are the Rho-family GTPases, which in immune cells are activated downstream of many immune receptors including the TCR, BCR, TLR, and chemokine receptors [see **Figure 1**) (reviewed in (7, 8)]. Upon ligand-receptor stimulation, conserved guanine nucleotide exchange factors (GEFs) are activated, which then convert Rho family members from the inactive GDP bound form to the active GTP bound form. Conversely, GTPase activating proteins (GAPs) stimulate hydrolysis of bound GTP to GDP, thus inactivating Rho members. In immune cells, the major Rho family GTPases include CDC42, RhoA, and Rac1 and Rac2. The primary essential Rho GEFs in immune cells include Dock8 (which activates CDC42), RhoGEF1 (which activates RhoA), Dock2 (which activates Rac1 and Rac2), and DEF6 (which activates CDC42 and Rac1/2) (**Figure 1**). RhoH, an additional atypical RhoGTPase that is not regulated by GTP exchange, is also present in hematopoietic cells and is believed to act in part by inhibiting the membrane association and activation of Rac-GTP. The importance of Rho GTPase signaling in human immunity has been highlighted by the identification of IEIs (“Rhopathies”) caused by loss-of-function variants in GEFs including Dock8 (9–16), RhoGEF1 (17), Dock2 (18, 19), DEF6 (20) as well as the GTPases CDC42 (21–23), Rac1 (24), Rac2 (25–29), and RhoH (30) [reviewed in (7, 8)]. Interestingly, differences in cell types and immune functions affected by each gene variant emphasizes the complexity of Rho signaling. For example, gene variants in the *ARHGEF1* gene encoding RHOGEF1 results in perturbed B cell homeostasis with a near absence of marginal zone and memory B cells, whereas homozygous mutations in the gene encoding DEF6 results in autoimmunity and hyperinflammation associated with impaired T cell responses. In mice, gene targeting studies further suggest that additional Rho pathway signaling molecules may be important in normal immune functions including the RhoGEFs Vav (Vav1, 2, 3), Tiam1 (T cell lymphoma invasion and metastasis 1), as well as Rho-GAPs including Bcr (Breakpoint cluster region), and RacGAP1 [see (31) for review]. Although Vav1 haploinsufficiency has been associated with common variable immunodeficiency (32), it remains to be determined whether LOF variants in the latter genes will be identified as causative in additional human IEIs.

The unique clinical consequences and immune cell types affected by loss-of-function Rho gene variants in PIDs are due in part to differential expression of Rho GEFs and Rho GTPases in specific immune cell types, as well as unique downstream target complexes activated by each Rho GTPase, which ultimately result in the control of F-actin polymerization, depolymerization, and contraction [see **Figure 1** and (7) for review]. Dynamic actin polymerization depends on the ability of monomeric G-actin to polymerize into filamentous form (F-actin), a process which is controlled by the 7-subunit actin-related protein 2/3 (ARP2/3). Specifically, the ARP2/3 complex stimulates the formation of new F-actin filaments at the sides of preexisting filaments from a pool of ATP- actin monomers bound by the actin binding protein Profilin. As F-actin filaments grow, associated membranes are pushed forward, while actin capping proteins antagonizes growth by binding the barbed end, resulting in termination of elongation and promotion of branching. In immune cells, actin nucleation by the ARP2/3 complex is primarily activated by WASp and WAVE, which are activated downstream of Rho-GTPases. WASp, in association with WIP (WASp interacting protein) is activated by GTP-bound CDC42, whereas the WAVE regulatory complex (WRC) is activated primarily by GTP-bound Rac1 or 2. The evolutionarily conserved WRC consists of Sra1 (Specifically Rac-associated protein 1, also known as CYFIP1/2 (cytoplasmic FMR1 interaction protein 1/2), Hem1 (Hematopoietic protein-like 1) or Hem2 (also known as Nck associated protein 1 (Nap1)), Abi1/2 (Abelson interactor 1 or 2), WAVE1 or 2 or 3, and HSPC300 (hematopoietic stem/progenitor cell protein 300, also known as BRICK1) (33, 34). Importantly, expression of the *NCKAP1l* gene encoding HEM1 is predominantly restricted to hematopoietic cells (35, 36), and *WAVE2 (* 37), although ubiquitously expressed, is the dominant member expressed in hematopoietic cells (34). In addition to activating the ARP2/3 complex, both Rac-GTP and CDC42-GTP lead to activation of the serine threonine kinase PAK1 (P21 associated kinase 1), which activates LIM kinase and further stabilizes actin polymerization by phosphorylating and preventing the actin binding protein Cofilin from severing ADP-bound actin and promoting actin depolymerization. Both Rac-GTP and CDC42-GTP also activate DIAPH3, which facilitates nucleation of ATP-bound actin, and likely other formins including the FMNL family (FMNL2 and FMNL3), which can generate a population of Arp2/3 complex-independent actin filaments in lamellipodia (38). Finally, RhoA-GTP also contributes to active actin polymerization by activating LIMK, and by facilitating actin contraction by activating ROCK1 or 2 (Rho-associated coiled-coil containing kinase), which inhibits myosin light chain phosphatase (MLCP) resulting in increased phosphorylation of myosin light chains (MLCs) which then promote myosin head binding to actin filaments, stimulating contraction. Similar to RhoGEFs and Rho GTPases, LOF variants in Rho effectors and NPFs including WIP (39), WASp (40), ARPC1B (ARP2/3 complex subunit 1B, a component of the ARP2/3 complex) (41–43) and HEM1 result in IEIs, consistent with the critical and non-redundant roles of the WASp and WAVE complexes in immune function. The clinical phenotypes, immune cells affected, and molecular regulation of LOF variants in WIP and WASp have been extensively reviewed elsewhere (44) and thus are not discussed here. However, the newly identified IEI due to LOF variants in HEM1 highlights the importance of understanding the regulation and downstream signaling regulated by the WRC and Hem1.

### Molecular regulation of the WAVE regulatory complex

**Figure 2:**
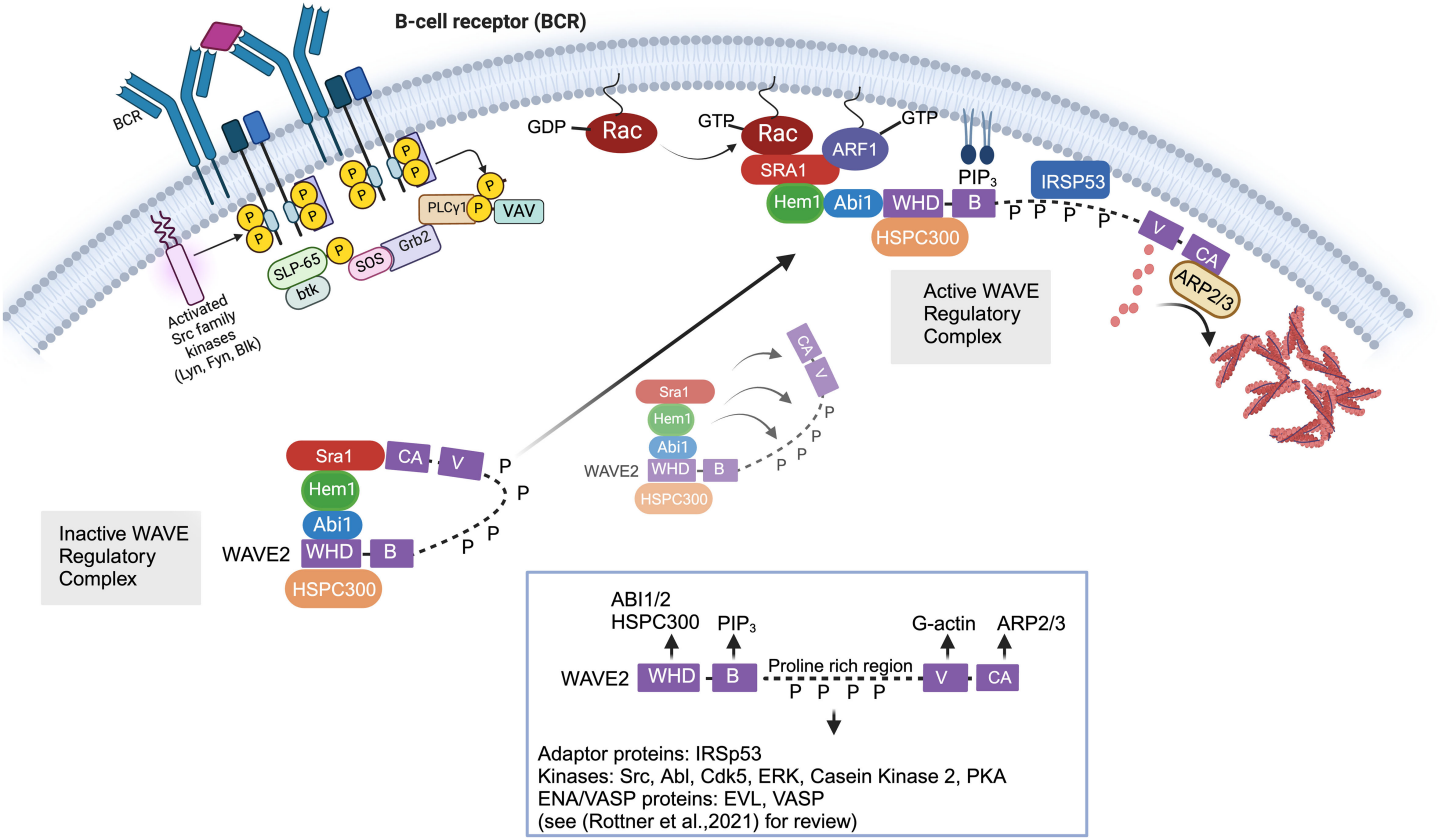
Cellular and molecular activation of the WAVE regulatory complex. In immune cells, the Wave Regulatory Complex (WRC) consists of Sra1, Hem1, Abi1/2, WAVE2, and HSPC300. The WAVE2 protein has multiple domains including an amino-terminal WAVE homology domain (WHD, which is important for interactions with other WRC members), a basic region (B) (which bonds phospholipids), proline rich region (PRR, which binds multiple SH3 containing proteins), a c-terminal Verprolin-homology domains (V), Cofilin domain (C), and acidic domain (A). The c-terminal V, C, and A domains (collectively known as the VCA domain) are central in binding G-actin monomers and ARP2/3 complex. The WRC exists basally in an autoinhibited “closed” state due to interaction of the “meander” and VCA regions of WAVE with Sra1. Stable activation of the WRC involves relocation of the WRC from the cytosol to the plasma membrane where it interacts with PIP3 via the WAVE basic region (B), IRSp53 via the WAVE PRR, and Rac-GTP and ARF1 via Sra1. These synergistic interactions collectively promote the allosteric release of the VCA domain from the “autoinhibiting” interaction with Sra1, allowing the VCA domain to interact with actin monomers and the ARP2/3 complex resulting in actin nucleation and formation of branched actin networks. In non-immune cells, it is known that multiple phosphorylation events additionally regulate the stability of WRC activation. For example, SH3 domain containing kinases such as Abl, Src, and Cdk5 interact with the proline rich region of WAVE via SH3 domains. Phosphorylation of WAVE2 by Abl kinase has been shown to be important for WRC-actin assembly and lamellipodia formation, and phosphorylation by Src or Cdk5 alters actin dynamics. However, we have not yet obtained a full understanding of the kinases that interact with and phosphorylate WAVE in immune cells. Of note, various transmembrane receptors may also contribute to WRC recruitment to (or its regulation at) the plasma membrane using a specific peptide motif called the WIRS (WRC Interacting Receptor Sequence) which binds a pocket on the surface of the WRC via Abi2 and Sra1 [not shown in Figure but reviewed in (45)].

The WRC is activated by a complex and highly regulated process involving release from autoinhibition; recruitment from the cytoplasm to plasma membrane via interactions with GTPases, phospholipids, membrane receptors, and kinases; and multiple phosphorylation events (**Figure 2**) [see (45) for review]. Structurally, WASp and WAVE family proteins are highly homologous and share Basic regions (B), proline rich regions (PRR), c-terminal Verprolin-homology domains (V, also known at the WASp Homology 2 (WH2) domain), Cofilin homology domains (C, also known as the central domain), and Acidic domains (A). The c-terminal V, C, and A domains (collectively known as the VCA (or WCA) domain) are central for binding G-actin monomers and the ARP2/3 complex. The WASp family proteins have a unique conserved amino-terminal WASP homology (WH1) domain which is important for protein-protein interactions [see (46) for review]. In contrast, the WAVE family proteins contain a conserved amino-terminal WAVE homology domain (WHD) which is important for interactions with other WRC subunits. Stoichiometry studies suggest that GTP-bound Rac directly binds two sites on Sra1 (47, 48), which binds Hem1/2 (33). Hem1/2 interact with Abi1/2 (34), which binds the WHD domain of WAVE and HSPC300. Crystal structures revealed the WRC is divided by subcomplexes consisting of a Sra1:Nap1(Hem1) dimer and a WAVE: Abi2:HSPC300 trimer (49). The WRC exists basally in an autoinhibited “closed” state (50) due to interaction of the “meander” and VCA regions of WAVE with Sra1 (**Figure 2**) (48, 49). Stable activation of the WRC occurs by a complex series of cytosolic biochemical events which involve relocation of the WRC from the cytosol to the plasma membrane where it interacts with PIP3 (phosphatidylinositol 3,4,5 triphosphate) via the WAVE basic region (B) (51), IRSp53 (insulin receptor tyrosine kinase substrate p53) via the WAVE PRR (52), and Rac-GTP and ARF1 via Sra1 (**Figure 2**). These synergistic interactions collectively promote the allosteric release of the VCA domain from the “autoinhibiting” interaction with Sra1, allowing the VCA domain to interact with actin monomers and the ARP2/3 complex resulting in actin nucleation and formation of branched actin networks. WAVE also interacts via the PRR with multiple SH3 domain containing proteins including kinases such as Abl (53, 54), Src, Cdk5 (cell division protein kinase 5) (55), MAPKs (56), and Casein kinase 2 (57), resulting in phosphorylation of the WRC at multiple sites. WRC phosphorylation regulates interactions with additional auxiliary proteins and promotes both stabilization and destabilization of VCA binding and WRC activation (57). For example, phosphorylation of WAVE2 by Abl kinase has been shown to be important for WRC-mediated actin assembly and lamellipodia formation (53, 54, 58), and phosphorylation by Src or Cdk5 alters actin dynamics (55). The PRR regions also interacts with Ena/VASP homology (EVH1)-domain-containing proteins, which enhance barbed end actin filament elongation and prevent capping proteins from binding (59). Additionally, it’s been recently realized that various transmembrane receptors may recruit the WRC to the plasma membrane using a specific peptide motif called the WIRS (WRC Interacting Receptor Sequence) which binds a pocket on the surface of the WRC via Abi2 and Sra1 [reviewed in (45)]. The importance of specific WIRS and kinase interactions/phosphorylations with the WRC has not been extensively studied in immune cells and hence is an important area of new investigation.

### The Hem family members have evolutionarily conserved functions

Whereas Hem1 is expressed almost exclusively in hematopoietic cells, the ortholog Hem2 (also known as NCK associated protein 1 (NAP1) encoded by the *Nckap1* gene) is more widely expressed in brain, heart, kidney, gastrointestinal tissues, liver, and skeletal muscle but is not expressed in hematopoietic cells (35, 36). These differences in expression are also reflected by disparate associations with human disease. For example, LOF variants in the *NCKAP1* gene encoding HEM2 in humans have been associated with neurodevelopmental disorders with features of autism (60, 61), whereas LOF variants in *NCKAP1L* encoding HEM1 are associated with immunodeficiency, hyperinflammation, and autoimmunity [discussed below and reviewed in (62)]. Hem Family members are evolutionarily conserved in *Arabidopsis* (Nap1), *Dictyostelium* (Nap1), *C.elegans* (GEX-3), and *Drosophila melanogaster* (KETTE). Although the structure of Hem family proteins does not contain any known domains, there are multiple hydrophobic regions and cysteine residues consistent with interactions with Sra1, as defined by crystal structures (63). Hem1 protein also contains multiple phosphorylation, acetylation, and ubiquitylation sites, although the significance of these modifications are not known.

Multiple immunoprecipitation (IP) and structural studies support the association of Hem1 protein with WAVE, Abi1, and Sra1. However, additional protein interactions have been noted. For example, IP studies using lysate from the human promyelocytic leukemia cells (HL-60) with anti-Hem1 antibodies identified interactions with additional polarity proteins including myosin light chain phosphatase, Rho-GAPs Myosin IXB, and Vps34 (a class III phosphatidylinositol-3-kinase (PI3K), all of which have been proposed to exclude myosin from the leading edge of migrating cells (34). These results suggest that Hem1 may have additional functions in regulating myosin-based contraction at the leading edge of cells. Another IP study using lysate from Jurkat T cells revealed interactions of Hem1, but not WAVE2, with mTOR and Rictor, components of the mTORC2 complex (64). The mTORC2 complex has various biological functions including regulation of cell proliferation, survival, and cytoskeletal organization in response to growth factor and nutrient stimulation. HEM1 deficient patient T cells and HEM1 knockdown in Jurkat T cells show reduced TCR induced phosphorylation of AKT at serine 473, a known mTORC2 phosphorylation site. Decreased BCR induced phosphorylation of AKT at serine 473 was also noted in HEM1 deficient human B cells (65), but not mouse B cells (66). These results suggest that HEM1 may have a WRC-independent role in human T and B cells in regulating mTORC2 enzymatic activity.

Analyses of conserved Hem family members across metazoans consistently support essential roles in F-actin dynamics, cellular morphogenesis, and differentiation. For example, disruption of NAP1 in the amoeba *Dictyostelium discoideum* resulted in impaired actin polymerization; small pseudopods; decreased adhesion, motility, and disrupted multicellular development (67, 68). Loss of GEX3 in the roundworm *Caenorhabditis elegans* resulted in impaired morphogenesis and migration (69), whereas disruption of Kette in the fruitfly *Drosophila melanogaster* led to disrupted actin cytoskeleton and defective axon projections and impaired myoblast fusion (70, 71). In mammals, RNAi mediated knockdown of *NCKAP1* (HEM2) in a melanoma cell line revealed that HEM2 is important for Rac-dependent formation of structures called lamellipodia in response to growth factor stimulation (72). Constitutive knockout of *Nckap1* in mice resulted in mid-gestation lethality due to neurulation defects, impaired neuronal differentiation, failed heart tube development, and delayed migration of endoderm and mesoderm (73).

## Murine models of Hem1 deficiency

### Hem1 is essential for coordinated immunity and prevention of autoimmunity in mice

**Figure 3:**
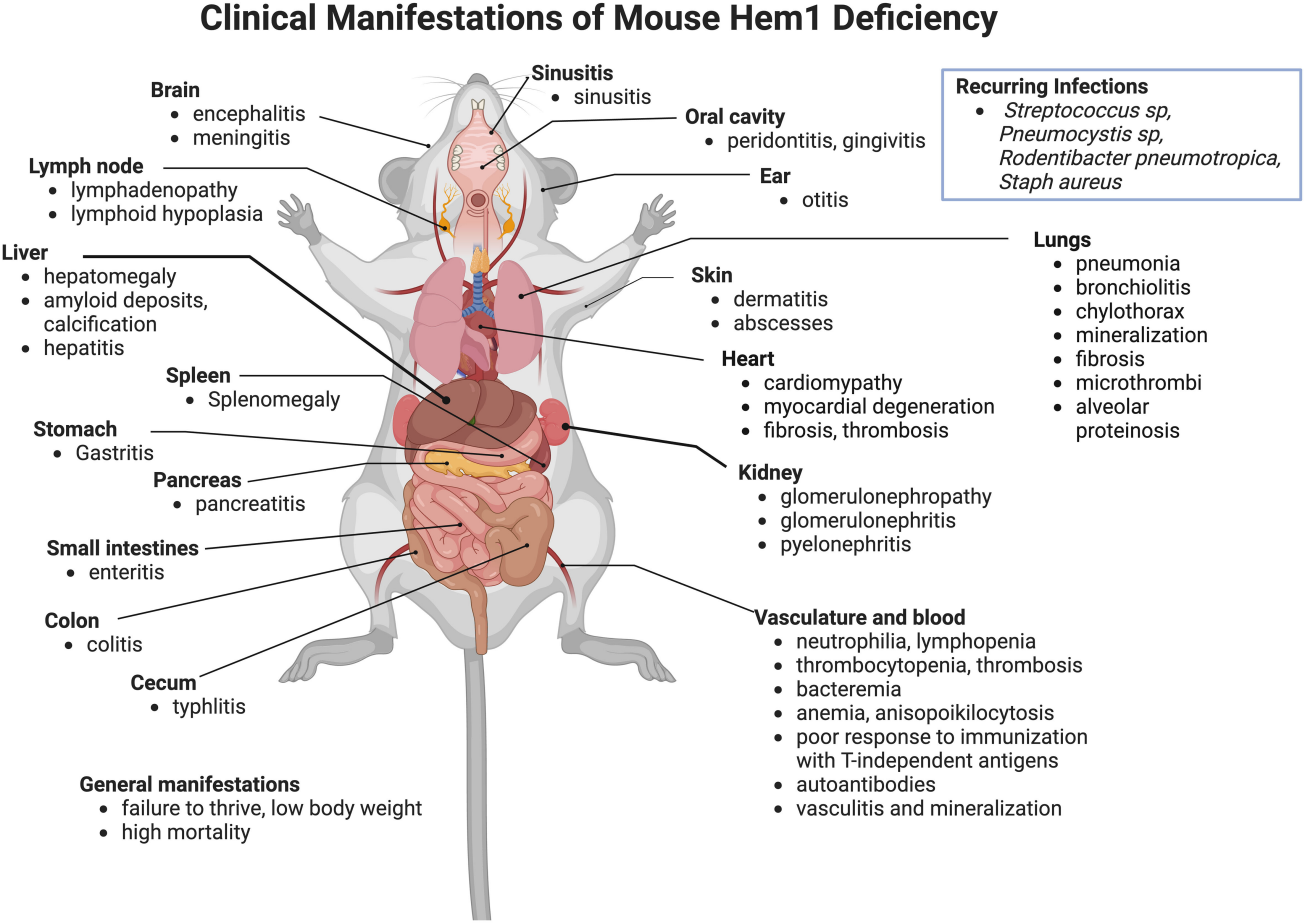
Clinicopathological features of Hem1 deficiency in mice. Shown are clinical and pathological findings noted in mice containing either a non-coding point mutation in Hem1 (Hem1^pt/pt^) (36, 77, 78), or Cre-mediated deletion of Hem1 (Hem1^-/-^) (65, 66, 74). As noted in **Figure 8**, the lesions and clinical findings noted in mice are remarkably similar to HEM1 deficient human patients. For example, despite being housed under Specific Pathogen Free (SPF) conditions, Hem1 deficient mice fail to thrive, exhibit low body weight, are more susceptible to opportunistic infections, and exhibit higher mortality. Inflammation is noted in multiple tissues including the gastrointestinal tract, kidney, pancreas, heart, lung, liver, skin, oral cavity, inner ear, sinuses etc. Signs of autoimmunity including increased autoantibodies were also noted.

The importance of Hem1 in the development and functions of hematopoietic cells and the immune system in mammals were first revealed in 2008 when *Park et al.* published the description of mice constitutively lacking Hem1 (36). Using an N-ethyl-N-nitrosourea (ENU) chemical mutagenesis strategy in mice to discover novel immune regulating genes, a mutant mouse line denoted NTB.1 (“no T no B”) was identified which had decreased T and B cells in peripheral blood samples due to a single non-coding point mutation in exon 13 of the *Nckap1l* gene which resulted in a premature STOP codon and termination at residue 445. Analyses of cell lysates from Hem1 deficient (*Nckap1^m1Iri^*, or *Hem1^pt/pt^*) thymocytes, peripheral T cells, and neutrophils demonstrated that the residue 445 variant resulted in the absence of Hem1 protein. *Hem1^pt/pt^* mice were notably smaller than their wildtype littermates and were significantly more susceptible to opportunistic infections. *Hem1^pt/pt^* mice presented with a spectrum of gross and histological pathology lesions including splenomegaly, hepatomegaly, dense amyloid accumulations containing foci of mineralization at the liver margins, and occasional diffuse inflammation of the thoracic pleura, heart, lungs, epididymis, and mesentery (36). Also noted were membranoproliferative glomerulopathy, diffuse lymphoid hypoplasia in lymphoid organs, myocardial fibrosis, amyloidosis, and pancreatic atrophy. Thymi were hypoplastic, consistent with altered T cell development and stress induced apoptosis (discussed below). Splenomegaly was characterized histologically as extramedullary hematopoiesis, which correlated with decreased myeloid, erythroid, and lymphoid progenitors in the bone marrow and increased progenitors and Lin^-^Sca1^+^cKit^-^ hematopoietic stem cells (HSCs) in the spleen and peripheral blood based on *in vitro* colony forming assays. Additional constitutive *Hem1^-/-^* models were subsequently generated (65, 74–76). In addition to the lesions noted above, pathological findings noted included hyperinflammation in liver, brain, sinus, stomach, pancreas, small and large intestines, cecum, trachea, esophagus, and skin (**Figure 3**). Despite being housed under Specific Pathogen Free (SPF) conditions, some opportunistic pathogens were noted at necropsy, which included *Streptococcus sp, Rodentibacter (Pasteurella) pneumotropica, Staphylococcus aureus*, and *Pneumocystis* sp. These results collectively suggested that Hem1 is critically important for optimal immunity to microbial pathogens and the prevention of hyperinflammation. As discussed below, the clinical and pathological manifestations of Hem1 deficiency in mice and humans are strikingly similar.

Immunological analyses of *Hem1^pt/pt^* mice, *Hem1^-/-^*, and mice conditionally deficient in Hem1 only in specific immune cell types are described in detail below categorized by immune cell type. Some findings were expected based on the known importance of actin in specific cellular processes, but also many unexpected findings were noted emphasizing the importance of detailed immunological, hematological, and pathological analyses.

### T lymphocytes

The *Hem1^pt/pt^* murine model demonstrated Hem1 deficiency in mice resulted in disruption of T cell development and function including activation, proliferation, and differentiation (36). *Hem1^pt/pt^* mice exhibited severe lymphopenia accompanied by a reduction in thymocyte cell number compared to WT mice (36, 74). Thymocyte development was impaired at the CD4^-^CD8^-^ double negative (DN) to CD4^+^CD8^+^ double positive (DP) stage of development, specifically at the DN3 to DN4 transition which is mediated by the formation of the preTCR. CD4, CD8, and γδ T cells are also markedly reduced in the periphery in *Hem1^pt/pt^* mice (36). Increased percentages of FoxP3^+^ regulatory T cells were present in the thymus and periphery. While the mechanism of impaired T cell development is not clear, *Hem1^pt/pt^* mice fail to thrive even in SPF animal facilities, and thus it is likely that the disruption in T cell development in the thymus is due in part to stress and cortisol-induced apoptosis rather than specific effects on differentiation (79, 80).

**Figure 4:**
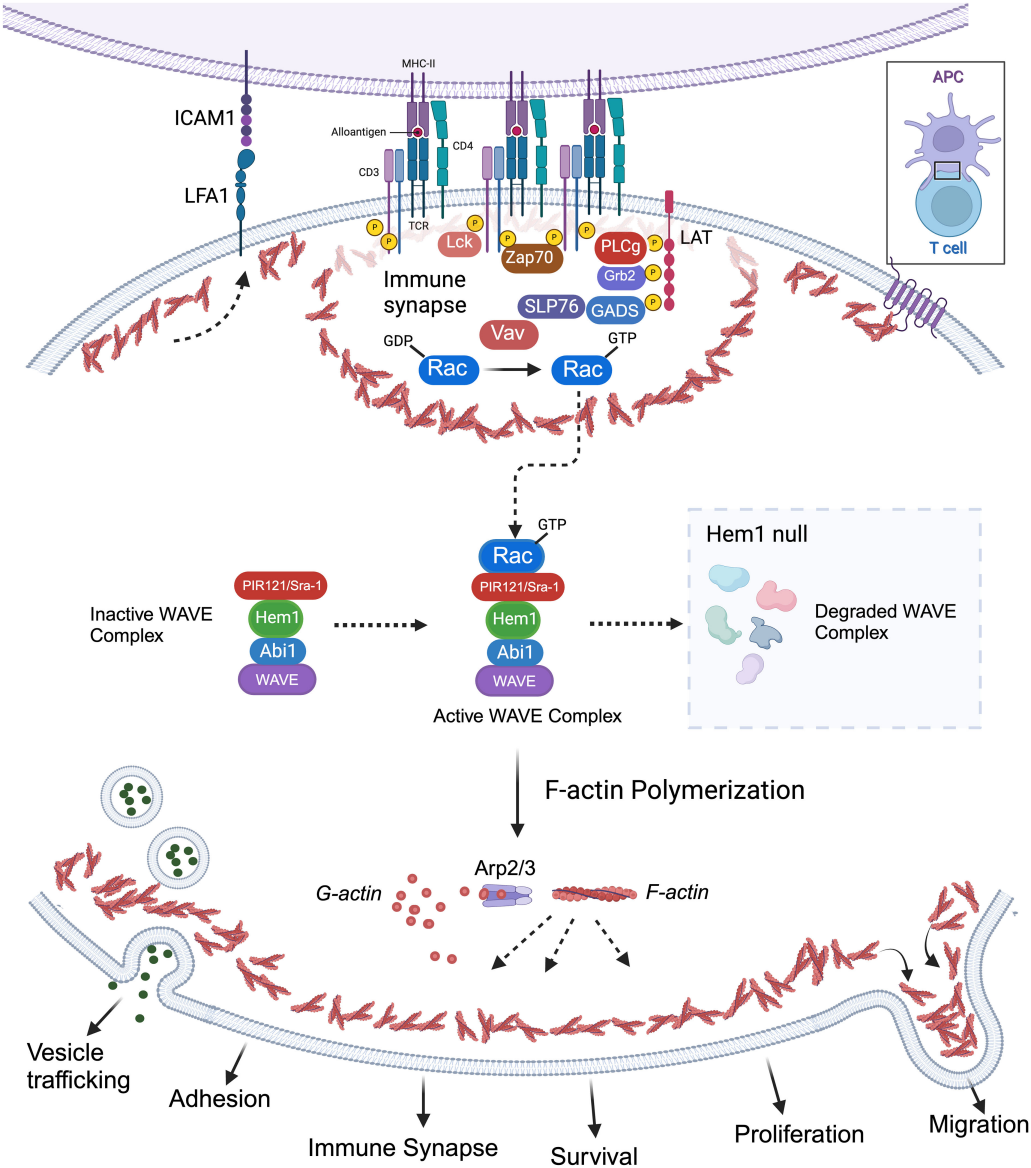
Importance of Hem1 and the WAVE regulatory complex in murine T Lymphocytes. The T cell receptor is (TCR) is activated following recognition of antigen displayed by an antigen presenting cell (APC), resulting in downstream activation of Rac from the Rho family of GTPases. GTP-bound Rac interacts with the WAVE regulatory complex (WRC) which facilitates F-actin polymerization via the ARP2/3 complex. Hem1 is a crucial component of the WRC and Hem1 deficiency results in destabilization and degradation of WAVE complex proteins, resulting in defective F-actin polymerization and branching in T cells. Branched actin has many important roles in T lymphocytes including formation of lamellipodia required for migration; integrin activation (e.g. LFA1 etc.) and focal adhesion formation for cell-cell adhesion; immune synapse formation required for APC interactions and concentrating T cell signaling molecules for optimal T cell activation; creating a cortical actin barrier to prevent inappropriate release of vesicles containing cytokines and other effector molecules; proliferation and survival.

Park et al. also discovered that loss of Hem1 resulted in impaired T cell activation and proliferation (36). In response to anti-CD3/CD28 activation, T cells from *Hem1^pt/pt^* mice had decreased expression of the activation marker CD69 and decreased proliferation as measured by CFSE staining. In addition, Hem1 deficient T cells and thymocytes had decreased F-actin polymerization as measured by phalloidin staining and had decreased actin capping at the immune synapse when stimulated with anti-CD3 coated beads. Integrin-mediated adhesion of *Hem1^pt/pt^* T cells to fibronectin was decreased following TCR stimulation (36). These results suggest Hem1 is essential for actin organization and adhesion of the immune synapse (**Figure 4**). No differences were found in proximal TCR signaling or Rac activation; however markedly decreased levels of WAVE proteins Wave2, Abi1, Abi2, and Sra1 were found in purified thymocytes and T cells via immunoblotting consistent with the observation that Hem1, and other WRC components, are necessary for the stabilization and/or translation of the WAVE complex proteins (34, 36, 71, 72, 81).

Hem1 deficiency was also shown to alter cytokine production potentially contributing to alterations in immune homeostasis. Stimulated *Hem1^pt/pt^* T cells produced normal levels of IL-2, IFN-γ, and TNF-α, however the pro-inflammatory cytokines IL-17 and IL-6 were markedly increased both *in vitro* and *in vivo* (36). To investigate whether Hem1 modulates Th17 differentiation, purified *Hem1^pt/pt^* naive T cells were expanded in Th1 and Th17 polarizing conditions. The absence of Hem1 did not appear to affect Th1 differentiation, however *Hem1^pt/pt^* T cells exhibited enhanced Th17 differentiation with an increased proportion of naive T cells expressing IL-17 following culture in Th17 polarizing conditions (36). The *Hem1^pt/pt^* murine model supports that *Hem1* plays an important role in T cell development, adhesion, immune synapse formation, cytokine (vesicle) release, and homeostasis secondary to disruption in F-actin and WAVE complex proteins (**Figure 4**).

Salzer et al. characterized a different mouse model of constitutive Hem1 deficiency generated via Cre/lox recombination that resulted in deletion of exons 4 and 5 within the *Hem1* gene (*Nckap1l^tm1.2Sixt^* or *Hem1^-/-^*) (65, 76). *Hem1^-/-^* mice suffered from autoimmunity characterized splenomegaly and glomerulonephritis similar to *Hem1^pt/pt^* mice. *Hem1^-/-^* mice had markedly increased levels of proinflammatory markers, including IL6, IFNγ and MRP8/14 (65), and had increased memory T cells similar to findings in human patients (64, 65, 82).

### B lymphocytes

**Figure 5:**
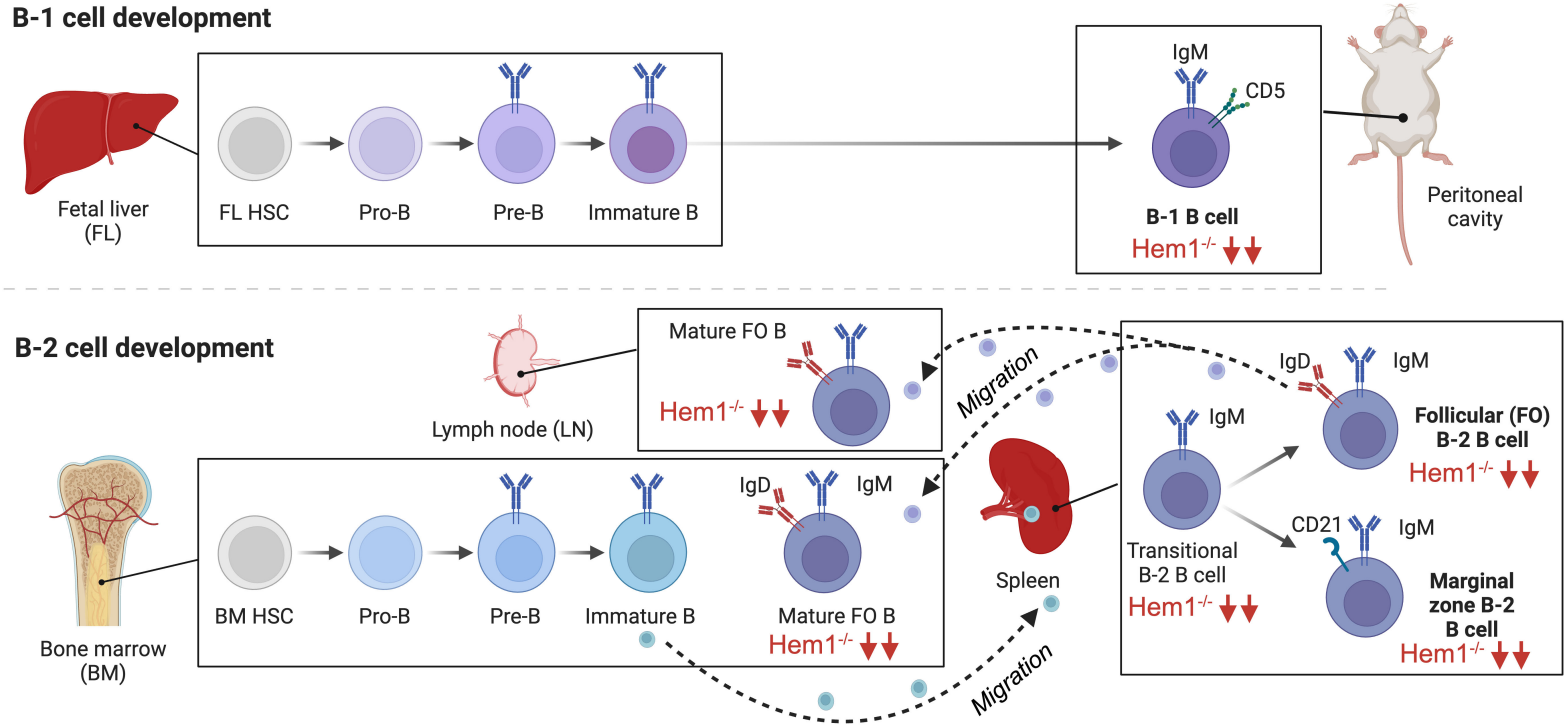
Hem1 regulates murine B lymphocyte development. B cells can be broadly divided into conventional B-2 B cells, which constitute the majority of B cells that interact with T follicular cells to produce specific antibodies, and fetal liver derived self-renewing innate-like B1a B cells, which are predominantly found in peritoneal and pleural cavities and produce natural antibodies. B2 B cells develop in the BM and fetal liver, where HSCs become committed pro-B cells, which have their Ig genes in germline configuration. Following Ig heavy chain gene rearrangement, the IgH protein is expressed at the surface of pre-B cells in conjunction with Igα and Igβ as the pre-BCR, which stimulates IgL chain rearrangement and formation of the BCR on immature B cells. Immature B cells which are deemed non-autoreactive migrate to the spleen where development continues through the transitional (T1,T2) stages, followed by differentiation into innate-like marginal zone (MZ) B cells or mature recirculating Follicular (FO) B cells, which migrate to LN and BM and produce specific antibodies. B1a B cells develop predominantly in the fetal liver and follow a similar developmental progression. B cell specific disruption of Hem1 in mice results in significant reduction of transitional B, MZ, FO, and B1 B cells in bone marrow, spleen, and lymph node tissues.

Analyses of *Hem1^pt/pt^* (*Nckap1l^m1.1Iri^*) and *Hem1^-/-^* (*Nckap1^tm1.1Iri^*) mice indicated that constitutive loss of Hem1 in all hematopoietic tissues resulted in a reduction of T and B lymphocytes in peripheral blood by at least 75% relative to WT mice (36, 66). *Hem1^-/-^* mice had a dramatic reduction in B cell numbers in the bone marrow, beginning with a reduction of the earliest pro-B and pre-B cell stages and culminating in a near-total absence of mature recirculating follicular (FO) B cells (**Figure 5**). A similar effect was observed in spleen, with *Hem1^-/-^* mice exhibiting reductions at all transitional stages (T0-T3), and extreme losses in mature FO and innate-like marginal zone (MZ) B cells. Peritoneal B1a, B1b, and B2 cells are also significantly depleted in these mice (65, 66).

In order to obtain better resolution on the cell-type specific effects of Hem1 deficiency in B cells, a conditional knockout *Hem1^fl/fl^Mb1Cre^+^* mouse model was created, whereby Hem1 was deleted only in B lymphocytes starting at the pro-B stage (66). Analyses of *Hem1^fl/fl^Mb1Cre^+^* mice revealed that B cell development proceeded relatively normally in the bone marrow up to the immature B cell stage (**Figure 5**). However, after immature B cells migrated to the spleen where development continues, transitional B (T0-T3), FO, and MZ B cells were all significantly reduced as was previously noted in constitutive *Hem1^-/-^* mice. The reduction of splenic FO B cells led to a reduction of mature recirculating FO B cells migrating back to the bone marrow and lymph nodes, tissues important for generating antibody responses to pathogens. Innate like-B1 B cells were also significantly reduced in *Hem1^fl/fl^Mb1Cre^+^* (66)(**Figure 5**). *In vitro* and *in vivo* migration assays also demonstrated that B cell specific loss of Hem1 led to poor migration capacity in response to chemokine stimulation, as has been noted in other immune cell types with WRC defects (66). These results suggest that Hem1 is important for the development of murine B cells in part by regulating cell migration and/or retention/survival in lymphoid tissues.

To test the functional consequences of Hem1 loss specifically in B cells, *Hem1^fl/fl^Mb1Cre^+^* mice were immunized with T cell independent (TI) or T cell dependent (TD) antigens and antibody responses were assessed (66). *Hem1^fl/fl^Mb1Cre^+^* mice failed to produce significant amounts of IgM antibodies against TI antigens such as NP-Ficoll and heat-killed (HK) *Streptococcus pneumoniae (Spn)*, likely due to reduced numbers of innate-like MZ and B1 B cells. Immunization with HK *Spn* also failed to protect of *Hem1^-/-^* and *Hem1^fl/fl^Mb1Cre^+^* mice following subsequent challenge with live *Streptococcus pneumoniae* whereas WT mice were completely protected. These results suggest that Hem1 is critically important for generation of protective antibody responses against TI antigens such as polysaccharides and lipopolysaccharides present in certain microbes.

Contrary to their impaired T-independent antibody responses, *Hem1^fl/fl^Mb1Cre^+^* FO B cells, which depend on T cells to provide costimulatory signal, subsequently produced more IgM and IgG2c antibody 3-4 weeks after immunization with TD antigens (66). Although no clinical differences were noted following infection of *Hem1^fl/fl^Mb1Cre^+^* and control mice with mouse adapted influenza virus (IAV), germinal center (GC) and plasma cell production were increased in Hem1 deficient mice, which was also noted following immunization of *Hem1^-/-^* mice with virus-like particles (66). *Salzer et al.* also found that constitutive disruption of Hem1 resulted in elevated GC B cells (65). These results suggest that TD antibody responses appear to proceed rather normally in the absence of Hem1 in a B cell specific manner.

Increased expression of IgG2c in C57BL6J mice or IgG1/3 in humans has been associated with increased autoimmunity. Class switching to IgG2c (IgG1/3 in humans), memory B cells survival, and PC differentiation (83) are induced by the transcription factor T-bet (84–88). A unique population of memory B cells expressing T-bet and CD11c denoted “age associated B cells (ABC)” are known to expand following chronic microbe stimulation and are elevated in autoimmune diseases such as SLE (89–94). Flow cytometric analyses of *Hem1^-/-^* and *Hem1^fl/fl^Mb1Cre^+^* mice revealed increased representation of ABC-like T-bet^+^ B cells, which was associated with increased IgM and IgG autoantibodies in sera following hybridization to autoantigen arrays containing 128 autoantigens (66). Increased anti-double stranded DNA autoantibodies were also noted in sera from *Hem1^-/-^* mice as early as 3 weeks of age (65). Purified B220^+^CD21^lo^CD23^+^ B cells from *Hem1^fl/fl^Mb1Cre^+^* mice activated *in vitro* expressed high levels of the T-bet target gene *Ifng*, and high levels of IFNγ signaling promotes GC formation and autoantibody production in mice and humans (95, 96). Importantly as discussed below, in two independent studies, 50% of human patients with PID due to LOF variants in Hem1 were found to have increased CD19^+^CD38^lo^CD21^-/lo^ ABC-like B cells. These results suggest that B cell specific expression of Hem1 is essential for normal B cell development and prevention of autoimmunity.

Alterations in the representation of T-bet^+^ B cells and increased autoantibody production suggested that B cell signaling may be disrupted in Hem1 deficient mice. The first indication that this may be the case was that surface IgM expression was decreased and IgD expression was increased on Hem1 deficient B cells throughout B cell development in the BM and periphery relative to control B cells (66). It had been previously shown that IgM, but not IgD, is downregulated in autoreactive B cells with excessive signaling (97, 98). Relative to IgM, IgD antigen engagement has lower signaling strength and increased expression of IgD at the expense of IgM has been proposed as a mechanism for B cells to escape BCR-mediated deletion during development and become tolerant to self-antigens. Indeed, B cells from *Hem1^fl/fl^Mb1Cre^+^* mice were also found to be more activated both basally and following BCR stimulation as measured by increased expression of CD25 and CD69, and increased Ca^2+^ influx (66). These results collectively suggest that B cell specific disruption of Hem1 resulted in increased activation, both basally and following BCR stimulation.

**Figure 6:**
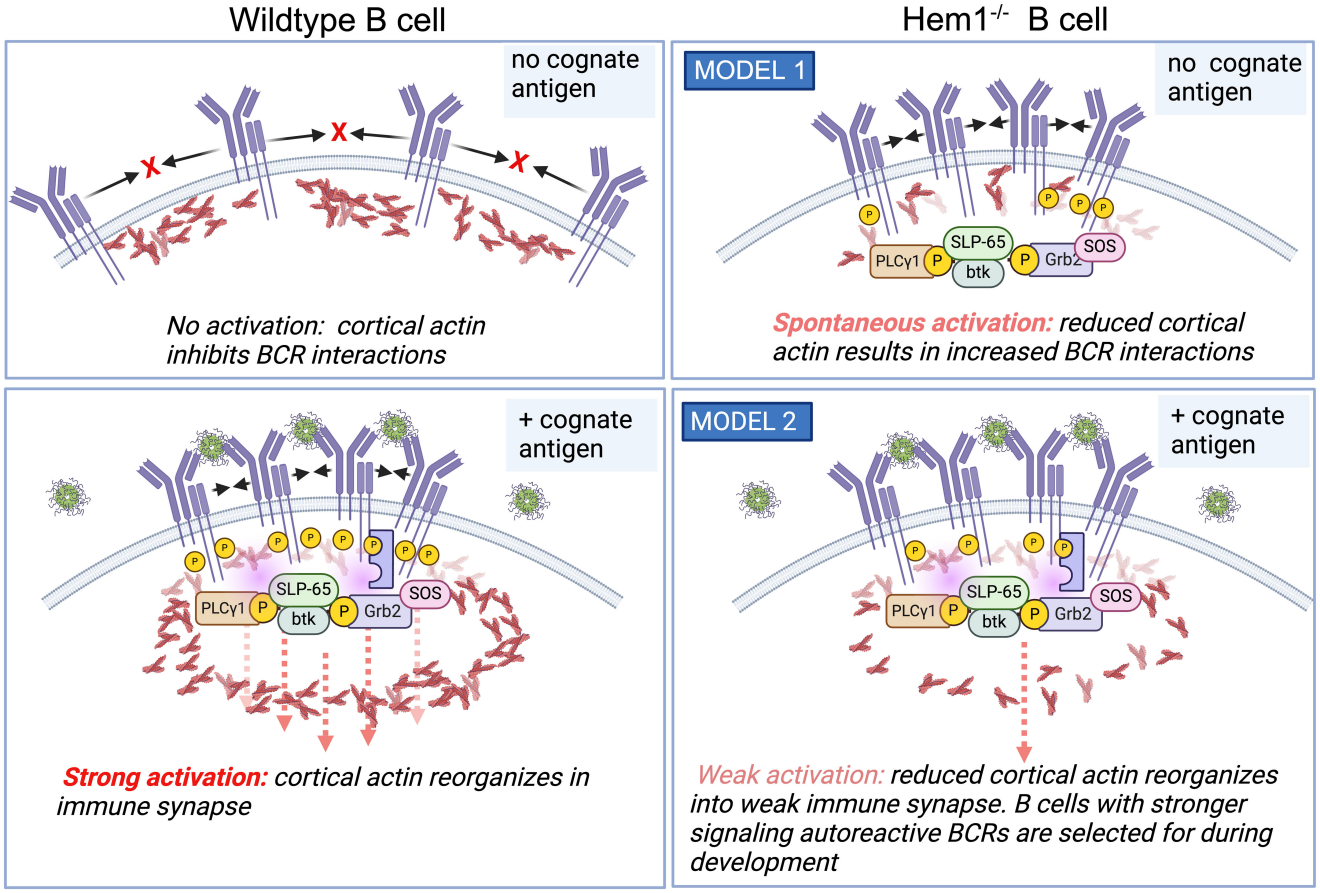
Hem1 deficiency results in B cell hyperactivation in mice. (*Top left*). In the absence of cognate Ag, BCRs are maintained at the cell surface in lipid-bound nanoclusters. (*bottom left*) Ag binding results in cortical actin depolymerization, resulting in increased BCR diffusion into microclusters and formation of the immune synapse, which then recruits and activates key signaling molecules resulting in B cell activation. (*top right*) One model is that in following disruption of Hem1, diminished cortical actin allows increased BCR diffusion and interactions in the absence of cognate antigen, resulting in basal or increased B cell activation. (*bottom right*) An alternative model is that disruption of Hem1 results in diminished immune synapse and microcluster formation, resulting in selection for stronger signaling autoreactive B cells during positive selection.

The exact mechanism for increased BCR signaling in Hem1 deficient B cells is unclear. In resting B cells, BCRs are maintained on the cell surface in lipid bound microclusters that are prevented from spontaneously interacting by networks of cortical actin (**Figure 6**, top left). Binding of cognate antigen results in subcortical actin depolymerization, formation of the immune synapse and increased BCR diffusion into microclusters, which recruit and activate key signaling molecules leading to B cell activation (**Figure 6**, bottom left) (99, 100). In one possible model, disruption of Hem1 results in reduced cortical actin, which enables multiple BCRs to spontaneously come into contact, crosslink, and initiate activation signaling in the absence of cognate Ag or in the presence of non-cognate Ag (**Figure 6**, top right) (66). Consistent with this model, treatment of primary B cells with actin-disrupting agents are sufficient to increase BCR diffusion and induce Ag independent, BCR- dependent Ca^2+^ signaling, Erk and AKT phosphorylation equivalent to BCR crosslinking (99, 101, 102). Another possibility is that Hem1-deficient B cells with reduced cortical actin are less able to effectively reorganize actin into the immune synapse and microclusters, resulting in reduced BCR signaling strength following interaction with cognate Ag (**Figure 6**, bottom right). This results in positive selection and survival of higher affinity autoreactive B cells during B cell development, leading to increased representation of autoreactive BCRs in the mature repertoire (65). Additional experimentation will be required to distinguish between these models.

### Neutrophils

**Figure 7:**
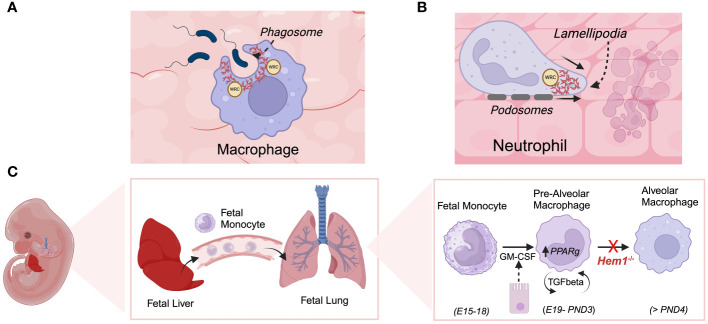
Hem1 regulates the development and/or functions of macrophages and neutrophils. **(A)** Engagement of phagocytic receptors upon binding to targets leads to activation of signaling pathways that stimulate formation of phagocytic cups, which contain adhesion molecules and are rich in branched actin foci, colocalized ARP2/3 and WRC. In the absence of Hem1, phagocytic cups fail to form properly and adhesion to targets are weak, leading to deficient phagocytosis. **(B)** Actin branching is important for forming lamellipodia, actin-filled protrusive structures which are critical for cell migration. The WRC accumulates and becomes anchored at the cell membrane, where it stimulates actin branching and forward driving force. Hem1 deficient macrophages and neutrophils are deficient in their abilities to migrate through complex matrices. **(C)** Alveolar macrophages (AMs) are a specialized subset that reside in lung airways. They develop via a unique process during fetal life, where fetal liver monocytes migrate to the fetal lung around embryonic day 15-18 (E15-18). In response to GM-CSF produced by bronchial epithelial cells around embryonic day 19 through post-natal day 3 (PND3), fetal monocytes develop into pre-alveolar macrophages. Thereafter, pre-AMs in response to autocrine TGF-β upregulate PPARγ which stimulates differentiation into mature AMs. In the absence of Hem1, AMs fail to develop efficiently, leading to an accumulation of debris and protein in airways (“alveolar proteinosis”).

Initial studies by *Weiner* et al. on Hem1 in the neutrophil cell line HL-60 and primary human neutrophils indicated that Hem1 protein is localized to the leading edge of polarized neutrophils and that the formation of Hem1 complexes are essential for chemotaxis by generating polarized actin polymerization, maintaining positive feedback loops that stabilize the leading edge, and preventing myosin phosphorylation at the leading edge (34, 103). Park et al. later showed that constitutive disruption of Hem1 in *Hem1^pt/pt^* mice resulted in significant neutrophilia (~ 25-fold increase), which correlated with reduced *in vitro* neutrophil migration in response to the chemoattractant fMLP, reduced F-actin polymerization, and lack of actin polarization toward the leading edge (**Figure 7B**), thus aligning with findings in primary neutrophils and HL-60 cells. *Hem1^pt/pt^* neutrophils were also deficient in phagocytosing fluorescent-conjugated beads and E. coli when stimulated with fMLP (36). The impaired phagocytosis by *Hem1^pt/pt^* neutrophils is consistent with the crucial role of F-actin polymerization in the formation of phagosome structures (**Figure 7A**).

Although the exact cause of neutrophilia in *Hem1^pt/pt^* mice is not known, the neutrophilic response correlated with increased numbers of proinflammatory Th17 cells, elevated serum IL-6 and IL-17, and heightened production of IL-1β, Mip-2, and KC from *in vitro*-stimulated macrophages (36). Increased IL-6 and IL-17 are known to potently stimulate the expansion and chemotaxis of neutrophils in a G-CSF dependent manner (104). These results suggest that a “feedback response” from impaired neutrophil migration into tissues combined with defective phagocytosis of tissue neutrophils by macrophages could result in increased production of IL-23 by tissue macrophages, leading to increased Th17 differentiation from naïve T cells and increased G-CSF stimulated neutrophil production (105). Consistent with this notion, *Hem1^pt/pt^* mice had a substantial increase in CFU-GM (colony-forming units-granulocyte and monocyte) and CFU-GEMM (CFU-granulocyte/erythroid/macrophage/monocyte) in the spleen and peripheral blood of compared to WT mice, suggesting that myelopoiesis was increased (36).

To further define the cell type specific roles of Hem1 in myeloid cells, *Hem1^fl/fl^* mice were generated and bred to *LysMCre* in the presence or absence of +tdTomato to delete Hem1 in the myeloid compartment and mark Cre expression (74). While *Hem1^fl/fl^LysMCre* mice were of normal size, they exhibited increased morbidity upon oropharyngeal challenge with Influenza A Virus (IAV) and intranasal *Streptococcus pneumoniae* (Spn). Notably, myeloid cell specific disruption of *Hem1^fl/fl^LysMCre* mice resulted in increased representation of immature neutrophils and macrophages in the BM suggesting that increased myelopoiesis is at least partially cell autonomous. Examination of neutrophils from *Hem1^fl/fl^LysMCre^+^* mice and *Hem1^fl/fl^ LysMCre+tdTomato* mice, using flow cytometry and fluorescent microscopy, revealed impaired F-actin polymerization in response to fMLP stimulation (74). Utilizing leukotriene B4 (LTB4) as a chemoattractant to assess migration, the absence of Hem1 led to reduced velocity and distance traveled, while the forward migration index (FMI) and directness were unaffected. The overall phenotype of Hem1 null neutrophils closely resembles that of neutrophils lacking the Rac-GEF Vav, characterized by defective adhesion, migration, and phagocytosis (106, 107).

### Macrophages

Initial analyses of *Hem1^pt/pt^* mice indicated that Hem1 null macrophages were significantly deficient in their abilities to phagocytose E. coli (36). This was confirmed in a study by *Stahnke* et al. using macrophages from an independently generated Hem1 knockout mouse model (*Nckap1l^tm1.2Sixt^*), whereby they showed that the phagocytosis of E. coli by *Hem1^-/-^* macrophages was diminished by over 80% compared to wild-type (WT) and *WASp^-/-^* macrophages. Notably, *Hem1^-/-^* macrophages failed to form conventional phagocytic cups; instead, particles sank into the cell covered by a loose membrane pocket (75). Time-lapse microscopy examination using IgG-opsonized sheep red blood cells (sRBCs) to test FcgR-triggered phagocytosis, revealed that *Hem1^-/-^* macrophages engaged multiple sRBC targets but frequently failed to fully phagocytose them (75).

*Hem1^-/-^* macrophages were also observed to be defective in adhesion and migration abilities. Engagement of the integrin CD11b around IgM/complement-opsonized beads was noticeably reduced in *Hem1^-/-^* but not *WASp^-/-^*, macrophages compared to WT macrophages (75). Defective adhesion to the phagocytic target became particularly evident during the late stages of envelopment, with *Hem1^-/-^* membranes displaying increased filopodia and gaps between the particle and membrane. In 3D collagen matrices, *Hem1^-/-^* macrophages exhibited poor performance in both migration velocity and directionality, which was associated with defects in lamellipodial protrusions that require assembly of a highly branched actin filament meshwork. Examination of leukocyte extravasation in the cremaster muscle revealed that blood vessels from mice lacking Hem1, but not WT or *WASP^-/-^* mice, were densely packed with myeloid cells. Additionally, the neighboring interstitial space was excessively populated with myeloid cells that had likely accumulated over time. Overall, constitutive loss of Hem1 in macrophages led to a decrease in both the frequency and efficacy of phagocytosis, impaired phagocytic cup formation, impaired integrin mediated adhesion, and poor migration capacity (75).

Alveolar macrophages (AMs) are a specialized macrophage subset that are abundant in the distal lung parenchyma and are the first subset to encounter incoming pollutants and pathogens and coordinate immune responses in the lung (**Figure 7C**). Resident AMs develop from fetal liver precursors that migrate to lung tissue and mature shortly after birth in response to GM-CSF and TGFβ, which together induce expression of *PPARG* (peroxisome proliferator–activated receptor-γ), a key transcription factor required for AM differentiation and functions. *Suwankitwat* et al. generated and analyzed *Hem1^-/-^* (*Nckap1l^tm1.1Iri^*) and *Hem1^fl/fl^LysM*Cre mice and found that Hem-1 deficiency resulted in the accumulation of excessive mucus and cell debris in the airways, leading to pulmonary alveolar proteinosis. This outcome was attributed to impaired development of AMs and reduced expression of *Pparg*. The remaining Hem1-deficient AMs exhibited a shift towards a proinflammatory immature phenotype, characterized by larger size, lower expression of CD11c, and higher CD11b. While the frequencies of monocytes and pre-AMs in the lungs were comparable between wild-type (WT) and *Hem1^-/-^* mice, the percentages and numbers of mature AMs were significantly reduced in lung tissue from *Hem1^-/-^* mice, indicating impaired maturation at the pre-AM to AM stage (74). AMs from adult *Hem1^fl/fl^LysMCre^+/+^* mice, upon LPS stimulation, expressed higher amounts of proinflammatory cytokine and chemokine mRNAs, along with lower levels of anti-inflammatory cytokine mRNAs compared to control AMs. Bronchoalveolar lavage fluid (BALF) from *Hem1^fl/fl^LysMCre^+/+^* mice infected with IAV showed increased levels of proinflammatory cytokines and chemokines such as granulocyte colony-stimulating factor (G-CSF), keratinocyte chemoattractant (KC), CCL2, and CCL3, indicating enhanced efforts to recruit inflammatory cells. Additionally, Hem-1-deficient AMs exhibited heightened production of proinflammatory cytokines and displayed impaired phagocytic activity *in vivo*, suggesting that the remaining AMs were functionally distinct and likely contributed to excessive inflammation during infection (74).

### Osteoclasts

Osteoclasts are a specialized macrophage-like cell that are derived from myeloid/monocytic lineage in the BM, and function to degrade normal or damaged bone as part of normal bone remodeling [see (108) for review]. During differentiation, hematopoietic progenitors proliferate, differentiate into preosteoclasts, which then fuse to become multinucleated mature osteoclasts. Using *Hem1^-/-^* (*Nckap1l^tm1Hro^*) mice, Krager et al. found that constitutive disruption of Hem1 resulted in a significant rise in bone mass due to impaired bone resorption in both male and female mice. Hem1 deficient osteoclasts developed normally but exhibited impaired fusion into multinucleated osteoclasts and diminished mitogen-activated protein kinase and tyrosine kinase c-Abl activity, resulting in reduced bone resorption. These findings highlight the crucial role of Hem1 in osteoclast differentiation and preserving normal bone mass (109, 110).

### Dendritic cells

To understand the importance of Hem1 in the development and functions of dendritic cells (DCs), Leithner et al. generated *Hem1^-/-^* mice (*Nckap1l^tm1.2Sixt^*) (76). Loss of Hem1 did not appear to affect the development of DCs. However, immature *Hem1^-/-^* DCs had reduced total F-actin levels and lacked lamellipodia. *Hem1^-/-^* DCs acquired a needle-like elongated shape with a rounded rear and sharply pointed front which contained actin filaments arranged linearly, demonstrating loss of the actin branches at the leading edge as the cause of the pointed cellular morphology. Once matured, *Hem1^-/-^* DCs formed thin filopodial extensions rather than the normal lamellipodial protrusions found in wildtype cells. Despite the abnormal protrusions at the leading edge, these DCs had a comparable mean overall cell body shape compared to wild type cells (111).

The distinct morphology of *Hem1^-/-^* DCs affected their ability to effectively migrate through their environments, showing only minimal response to chemotactic gradients. *Hem1^-/-^* DCs were unable to migrate to lymph nodes from the peripheral tissues and were defective in interstitial crawling and intravasation. The loss of lamellipodia, as an exploratory sensor, also affected their abilities to navigate through high-density, complex environments. However, the linear shape of *Hem1^-/-^* DCs allowed increased displacement speed and directional persistence compared to control cells.

In addition to affecting the intrinsic activity of DCs, loss of Hem1 also affected cell-to-cell interactions with other leukocytes due to decreased overall actin content at immunological synapses. *Hem1^-/-^* DCs activated only a small fraction of T cells compared to control cells despite expressing equal levels of surface MHCII and co-stimulatory ligands (76, 112). Surprisingly, Hem1 deficient mouse DCs demonstrated increased contact time with T cells due to increased ICAM-1-integrin mediated cell adhesions which resulted in fewer T cell:DC interactions and fewer T cells activated overall (112). T cells activated by *Hem1^-/-^* DCs did not show differences in proliferation or IL-2 production compared to T cells activated by control DCs.

### Hematopoietic stem cells

Analyses of *Hem1^pt/pt^* mice revealed that loss of Hem1 resulted in greatly enlarged spleens due to extramedullary hematopoiesis (36). As mentioned above, this correlated with decreased myeloid, erythroid, and lymphoid progenitors in the bone marrow and increased progenitors and Lin^-^Sca1^+^cKit^-^ hematopoietic stem cells (HSCs) in the spleen and peripheral blood. These results are consistent with increased mobilization of HSCs and progenitors (HSC/Ps) from the BM to peripheral blood and spleen, perhaps due to impaired adhesion and retention in the BM. Consistent with this hypothesis, *Hem1^pt/pt^* HSC/Ps failed to compete with WT HSCs/Ps following competitive bone marrow transplantation assays into irradiated syngeneic hosts, even when transplanted at a 1:10 WT: *Hem1^pt/pt^* HSC/Ps ratio (36).

An additional study by *Shao et al.* found that loss of the WRC secondary to deletion of *Hem1* in *Hem1^-/-^* mice (*Nckap1l^tm1Hro^*) led to failure of engraftment of fetal liver HSCs into bone marrow and subsequent premature exhaustion of neonatal bone marrow hematopoietic stem cells (113). *Hem1^-/-^* mice underwent marrow fibrosis and hematopoietic failure within 6-8 weeks. However, failure of engraftment was not due to defects in cell movement, such as chemotaxis, bone marrow homing, or adhesion. Instead, after arriving in the fetal bone marrow, *Hem1^-/-^* fetal liver hematopoietic stem cells underwent apoptosis secondary to loss of the intrinsic survival signaling by c-Abl. Rescue of c-Abl expression in *Hem1^-/-^* fetal liver cells restored the ability of these cells to engraft.

### Platelets and anemia

Analyses of peripheral blood samples from *Hem1^pt/pt^* mice indicated that loss of Hem1 resulted in microcytic, hypochromic regenerative anemia, which was associated with increased RBC progenitors and altered red blood cell morphology including anisocytosis (variation in RBC size), echinocytosis (contain evenly distributed pointed projections), and poikilocytosis (abnormally shaped RBCs). *Hem1^pt/pt^* RBCs were more fragile relative to WT RBCs as measured by increased percentage of lysis in response to hypotonic NaCl (36). Further analyses of *Hem1^pt/pt^* mice revealed that loss of Hem1 did not disrupt erythropoiesis but resulted in reduced RBC lifespan and increased fragility secondary to decreased representation of essential erythrocyte membrane skeletal proteins and altered phosphorylation of essential RBC junctional proteins, perhaps secondary to disruption of the actin cytoskeleton (77).

An additional study using *Hem1^-/-^* mice (*Nckap1l^Tm1.2Sixt^*) confirmed that loss of Hem1 resulted in irregular RBC morphology, but additionally identified aberrant platelet aggregates with increased total platelet counts and sizes (75). Interestingly, platelets lacking *Hem1* expressed similar levels of the platelet integrin α_IIb_β_3_, and were not completely devoid of protrusions or lamellipodia. However, *Hem1* null platelets had significantly reduced activation of α_IIb_β_3_ integrin in response to stimulation, affecting their spreading behavior and demonstrating a defect in integrin-mediated adhesion. This resulted in smaller thrombi with reduced volume when compared to WT platelets in a flow adhesion assay. These results suggest that loss of Hem1 has demonstrable effects on both RBC and platelet behavior.

## Human disease associated with Hem1 variants

### Loss-of-function variants in Hem1 result in primary immunodeficiency disease, hyperinflammation, and autoimmunity in humans

**Figure 8:**
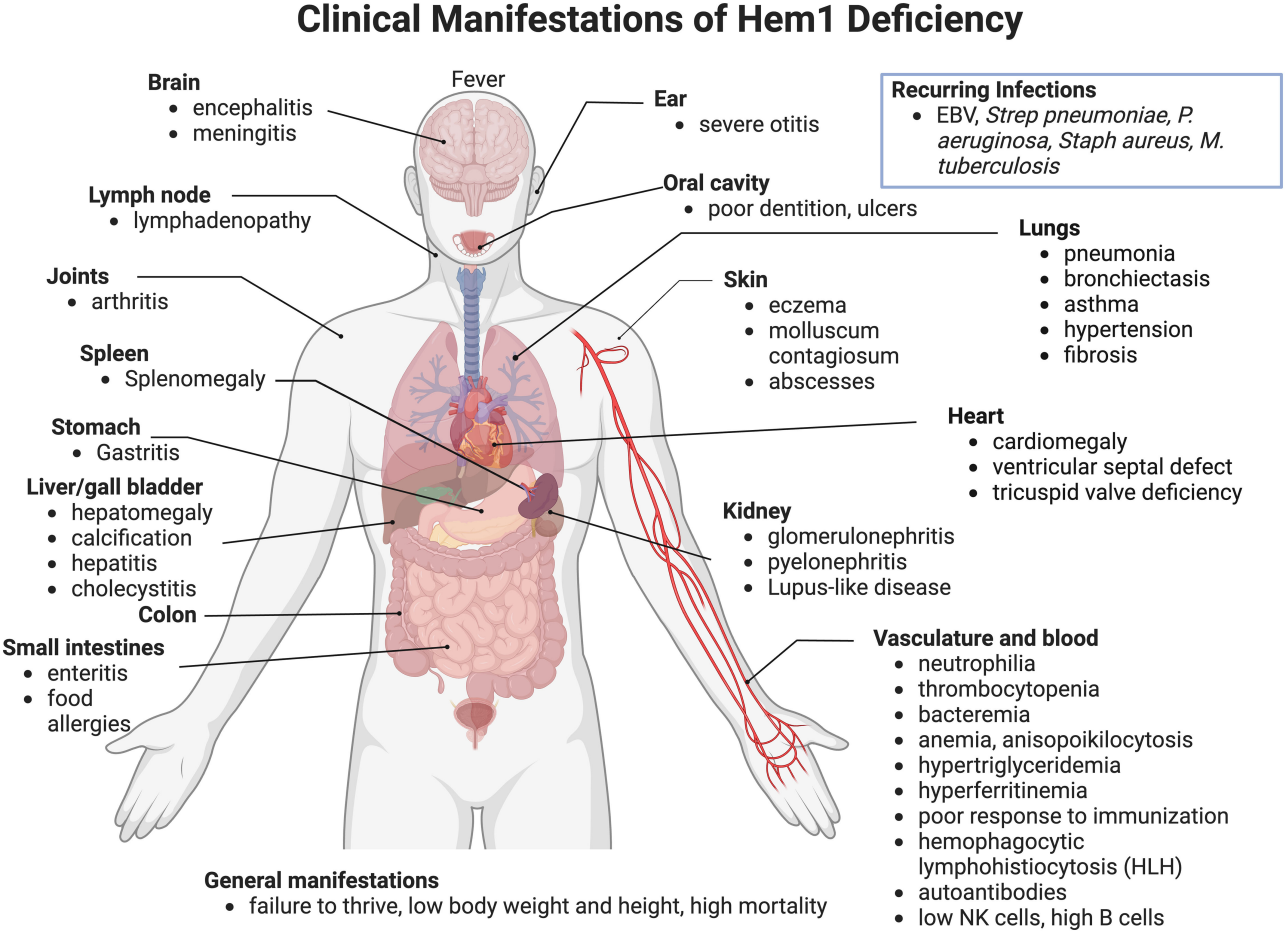
Clinical and pathological manifestations of Hem1 deficiency in humans. Shown are clinical and pathological findings in nine patients with loss of function mutations in Hem1 (62, 64, 65, 82). As noted in **Figure 3**, the lesions and clinical findings noted in Hem1 deficient patients are remarkably similar to murine models. Hem1 deficiency results in a syndrome characterized by immunodeficiency as well as hyperinflammation and autoimmunity. Although clinical presentations were varied among patients, all patients presented with recurrent infections including otitis media, skin abscesses and pneumonia. Systemic inflammation is also common resulting in hepatosplenomegaly and lymphadenopathy in several patients. Few patients also have autoimmune disorders resulting in autoantibodies, similar to what was seen in *Hem1^-/-^* mice models (36, 65, 66).

In 2020, nine pediatric patients from seven different lineages were identified with primary immune disorders (PID) secondary to loss-of-function mutations in the *NCKAP1L* gene encoding the Hem1 protein. HEM1 deficient patients were shown to have improper actin polymerization and networks leading to a clinical syndrome characterized by recurrent infections and autoimmunity (64, 65, 82) (**Figure 8**) [see (62) for review]. The clinical picture in the patients identified so far varies; however most suffered from recurrent infections and hyperinflammation resulting in a high mortality rate at a young age. In addition to immunodeficiency, more than half of the patients identified exhibited atopic or autoimmune diseases and some patients had disease involving hyperinflammation and lymphoproliferation similar to hemophagocytic lymphohistiocytosis (HLH) (64, 82).

Most patients presented within their first year of life with an average age of onset of 14 months (Range: 1.5 months - 5 years). Similar to Hem1 deficient mice, a hallmark of clinical disease of Hem1 deficiency seen throughout all patients was recurrent bacterial and viral infections, including otitis media, upper respiratory infections, pneumonia with complications of bronchiectasis, and skin infections. Several patients experienced poor seroconversion following immunization or infection with one patient contracting tuberculosis despite being vaccinated (82). In addition to immunodeficiency, most patients also suffered from hyperinflammation resulting in hepatosplenomegaly, lymphadenopathy, and hyperimmunoglobulinemia (62, 64, 65, 82). Atopic disease is also a common finding with several patients experiencing asthma or asthma-like symptoms (64). Three patients met criteria for hyperinflammation and lymphoproliferation similar to hemophagocytic lymphohistiocytosis (HLH) (64, 82). Two patients also experienced autoimmune diseases including signs consistent with systemic and nephrotic lupus (64, 65, 82). Other clinical features seen with Hem1 deficiency include developmental abnormalities, including pectus carinatum, ventricular septal defect, and poor dentition (64). Two patients suffered from failure to thrive with low height and weight (64, 65).

Hem1 deficiency results in a multitude of clinical syndromes, most of which can be attributed to dysfunction of immune homeostasis. Diagnosis of actin-related inborn errors of immunity (IEIs) such as HEM1 deficiency, can be challenging due to their clinical heterogeneity. Patients suspected of HEM1 deficiency based on their clinical presentation may undergo initial immune testing, such as immunoblotting, T and B cell activation studies, and neutrophil migration assays (62). Targeted gene sequencing panels allow for screening of already characterized IEI genes, such as HEM1, improving early diagnosis and medical management (3). Most patients received some form of immunomodulatory treatment, including corticosteroids, cyclosporine, sirolimus, mycophenolate mofetil, and rituximab (64, 65, 82). Additional common treatments among patients included antibiotics for opportunistic bacterial infections and asthma treatments such as bronchodilators (64, 65, 82). One patient had also been treated with IgG replacement therapy for pulmonary disease (64).

Immunophenotyping of Hem1 deficient patients yielded variable results, echoing the differences in clinical disease manifestations seen in patients, and concurrent treatments with immune modulating drugs. For example, some patients had normal numbers of CD4^+^ and CD8^+^ T cells (64, 65), whereas other patients had increased numbers of CD8^+^ T cells with a low CD4:CD8 ratio (64, 82). An inverted CD4:CD8 ratio was seen in *Hem1^pt/pt^* mice as well (36). Two unrelated patients had increased double-negative (DN) T cells (64); increased DN T cells are a hallmark of autoimmune lymphoproliferative syndrome (114). Some patients also had a relative increase in γδ T cells compared to αβ T cells, also seen in murine models (36, 65). Although patients had low to normal percentages of T regulatory cells (64, 82), *Hem1^pt/pt^* mice had increased percentages of FoxP3^+^ regulatory T cells (36). Decreased naïve T cells with increased central and effector memory cells was a consistent hallmark of Hem1 PID in humans (62, 64, 65, 82). Similar alterations in the naive and memory T cell populations were also seen in *Hem1^-/-^* mice (65).

Impaired T cell activation was another consistent feature of Hem1 deficient humans and mice, as evidenced by decreased upregulation of activation markers CD69 and CD25 (36, 64, 65, 82) and decreased proliferation (36, 64, 65) following TCR stimulation *in vitro.* T cell activation is highly dependent on actin polymerization for immune synapse formation, and likewise Hem1 deficient patient T cells had reduced accumulation of F-actin at the synapse and decreased cells spreading and lamellipodium formation at the synapse (64, 65, 82). T cells from *Hem1^pt/pt^* mice similarly had decreased F-actin polymerization following stimulation as well as decreased actin capping after stimulation with anti-CD3 coated beads (36). Castro et al. showed patient T cells exhibited an exhausted phenotype, and these patients had more autoimmune and HLH-like manifestations (82).

With respect to B cells, Hem1 deficient patients had normal to increased numbers of CD19^+^ B cells (64, 65, 82) in contrast to reduced B cell numbers in murine models with constitutive loss of Hem1 (36, 65, 66). In addition, patients had variable changes in naive and memory subsets. Castro et al. Identified a slight increase in the percentage of naive cells associated with a decrease in the percentage of memory B cells (82), while Salzer et al. found no difference in the percentages of naive, memory, and transitional B cells compared to age-matched normal ranges (65). Interestingly, patients had increased CD19^+^CD38^lo^CD21^lo^ (CD21^lo^) memory B cells, a subset of B cells associated with hyperinflammatory diseases (65). CD21^lo^ B cells have been described in several diseases associated with chronic inflammation or autoimmunity, and commonly also express CD11c, similar to the ABC-like B cells found in aged mice and SLE murine models (89–94). *Hem1^-/-^* and *Hem1^fl/fl^Mb1Cre^+^* mice revealed increased representation of ABC-like T-bet^+^ B cells similar to human patients (66). Three patients had autoantibodies associated with autoimmune disease, and likewise *Hem1^-/-^* and *Hem1^fl/fl^Mb1Cre^+^* mice had autoantibodies against several autoantigens (62, 64–66, 82). Patients also had variable immunoglobulin abnormalities with several patients having elevated levels of IgE particularly in patients with atopic disease or asthma (64, 82). Elevated IgG was also found in about half of patients with Hem1 deficiency (64, 82). Consistent with the findings in patient T cells, patient B cells also displayed decreased cell spreading and decreased lamellipodia formation at the immune synapse upon stimulation with IgM (65), as was seen in *Hem1^pt/pt^* mice (36).

Although human patients lacked the marked neutrophilia found in both the constitutive *Hem1^pt/pt^* and conditional *Hem1^fl/fl^LysMCre* murine model, both patient and murine Hem1 deficient neutrophils had significant migration deficits secondary to disruption of F-actin polymerization (36, 64, 74). Neutrophils from *Hem1* deficient patients exhibited reduced velocity and directional persistence when migrating in chemokine gradients (64). Similarly, neutrophils from *Hem1^pt/pt^* had decreased migration and *Hem1^fl/fl^LysMCre* mice had decreased migration and velocity as described previously (36, 74).

### Other clinical features associated with alterations in Hem1 expression

#### Inflammatory bowel disease

In a genome wide association study (GWAS) to identify genes that modulate networks associated with inflammatory bowel diseases (IBDs) in humans, *Peters et al.* identified *NCKAP1L* as a key driver gene (115). Using *Hem1^pt/pt^* mice lacking *Nckap1l* expression to test IBD association (36), the authors found that Hem1 deficiency did not cause spontaneous colitis, but increased susceptibility to intestinal inflammation in the dextran sulfate sodium (DSS) mouse model of colitis. Hem1 deficient mice had more severe weight loss and endoscopy scores after DSS treatment compared to their wild-type littermate controls. CD4^+^ T cells of the intestinal lamina propria of *Hem1* deficient mice had significantly increased IL-17A and IFNγ production while there were increased CD64^+^ macrophages and CD103^+^CD11b^+^ intestinal DCs and reduced CD11b^+^ single-positive DCs (36, 115). Increased IL17 and IFNγ have previously been associated with pathology in Crohn’s disease (116, 117), and naïve T cells from *Hem1^pt/pt^* mice show increased differentiation under Th17 polarizing conditions (36). These results suggest that dysregulation of HEM1 has a key role in IBD in addition to IEIs and autoimmunity, perhaps by modulating IL-17.

#### Chronic lymphocytic leukemia

In another gene expression analysis, *NCKAP1L* was found to be significantly overexpressed in B-cell chronic lymphocytic leukemia (CLL) cells expressing high CD38, a cell marker associated with poor clinical outcome. High levels of HEM1 expression correlated with more severe disease progression and shorter time to treatment. Interestingly, downregulation of HEM1 in these CLL cells increased their susceptibility to chemotherapeutic-mediated killing (118). These results suggest a potential involvement of the WRC and HEM1 in hematopoietic cancer invasiveness and/or survival.

### Challenges and future directions

Given the importance of the actin cytoskeleton in the immune system, the numerous players involved in the regulation of actin dynamics, and advancement in next generation sequencing technologies, it is not surprising that novel “actinopathies” are being discovered at a rapid rate. This has created significant challenges and opportunities, including defining the biological and functional consequences of gene disruptions over a vast array of immune cells, defining the roles of actin regulation at the cellular and molecular level, and developing and implementing gene specific therapies. Because, Hem1 is expressed almost exclusively in hematopoietic cells, it is possible that Hem1 disruption has some impact on virtually all hematopoietic cells. The severity of disease in Hem1 deficient children highlights the urgency to define the cellular and molecular consequences of HEM1 variants in all hematopoietic cells with the end goal of discovering novel therapies and/or combinations of existing therapies to improve clinical outcomes. Some additional important areas of investigation include:

(1) How is the WRC and Hem1 regulated in immune cells? The majority of studies regarding the regulation of WRC activation were not specifically performed in immune cells. For example, it would be interesting to know how SH3 containing proteins in lymphocytes including kinases (such as Lck, Fyn, Lyn, BTK, Itk), adaptor proteins (e.g. Grb2, GADS, etc.), and phospholipase signaling molecules (e.g. PLCγ) interact with the proline rich region of WAVE, and how this controls WRC activation and stability.
(2) Does Hem1 modulate progression of hematopoietic cancers? High expression of WRC components have been associated with various types of cancers including breast (WAVE2, WAVE3, ABI1, NAP1), ovarian (WAVE1, ABI1), prostate (WAVE1, WAVE3, ABI1), colorectal (WAVE2, WAVE3), liver (WAVE2, WAVE3), and lung (WAVE2) and are associated with tumor invasiveness and poor prognosis [see (119) for review]. Given that high Hem1 is associated with poor clinical outcome in CLL, it would be important to understand how high Hem1 expression moderates cancer progression at the cellular level, whether it be facilitation of invasion, increased adhesion of cancer cells into chemoprotective niches, and or adaptation to less hospitable environments (120). Dysregulation of Hem1 may also have a role in other hematopoietic cell cancers, and/or other novel mechanisms.
(3) What additional cellular functions are controlled by Hem1? It has been well characterized that the ARP2/3 complex is important for functions such as motility, morphogenesis, and phagocytosis. However, emerging evidence suggest that the ARP2/3 complex may control specific stress and nuclear responses such as autophagy, apoptosis, DNA repair, and chromosome dynamics [reviewed in (121)]. Actin is found in a variety of chromatin remodeling complexes and histone acetyltransferases and is hypothesized to maintain higher order chromatin structure. It would be informative to understand whether these and other cellular and molecular functions may be disrupted in immune cells from Hem1 deficient humans and mice.
(4) Modeling Hem1 deficiency in murine models. Given the limited number of human patients and concurrent treatments with immune-modulating drugs, it is critical to continue utilizing murine models to understand the cellular and molecular consequences of Hem1 deficiency on individual immune cell types, protective immunity, and predisposition to autoimmunity. Hem1 deficient mouse models exhibited remarkable clinical and pathological similarities to their human counterparts including increased susceptibility to bacterial and viral infections, hyperinflammation in multiple tissues and organs (hepatitis, enteritis, colitis, otitis, endocarditis, bronchiolitis, pneumonia etc), atopic diseases (eczema, etc), hepatosplenomegaly, thrombosis, autoimmunity (Lupus like disease, glomerulonephrosis, increased autoantibodies, etc), and failure to thrive overall. Comparative studies in mice and human samples will help elucidate the molecular and cellular consequences of Hem1 disruption in individual immune cell types, and the coordination of Hem1 deficient immune cells in overall protective immunity.

## Author contributions

AC: Conceptualization, Data curation, Formal Analysis, Investigation, Methodology, Project administration, Supervision, Validation, Visualization, Writing – original draft, Writing – review & editing. JT: Conceptualization, Data curation, Formal Analysis, Investigation, Methodology, Validation, Visualization, Writing – original draft, Writing – review & editing. NS: Conceptualization, Data curation, Formal Analysis, Investigation, Methodology, Validation, Visualization, Writing – original draft, Writing – review & editing. AA: Conceptualization, Formal Analysis, Investigation, Methodology, Visualization, Writing – original draft, Writing – review & editing. BI: Conceptualization, Data curation, Formal Analysis, Funding acquisition, Investigation, Methodology, Project administration, Resources, Supervision, Validation, Visualization, Writing – original draft, Writing – review & editing.
